# Mesenchymal Stem Cell Exosomes as Immunomodulatory Therapy for Corneal Scarring

**DOI:** 10.3390/ijms24087456

**Published:** 2023-04-18

**Authors:** Hon Shing Ong, Andri K. Riau, Gary Hin-Fai Yam, Nur Zahirah Binte M. Yusoff, Evelina J. Y. Han, Tze-Wei Goh, Ruenn Chai Lai, Sai Kiang Lim, Jodhbir S. Mehta

**Affiliations:** 1 Tissue Engineering and Cell Therapy Group, Singapore Eye Research Institute, Singapore 169856, Singapore; 2 Ophthalmology and Visual Sciences Academic Clinical Program, Duke-NUS Medical School, Singapore 169857, Singapore; 3 Corneal and External Diseases Department, Singapore National Eye Centre, Singapore 168751, Singapore; 4 Department of Ophthalmology, University of Pittsburgh, Pittsburgh, PA 15213, USA; 5 Institute of Medical Biology & Institute of Molecular and Cell Biology, Agency for Science, Technology and Research (A*STAR), Singapore 138648, Singapore; 6 School of Materials Science and Engineering, Nanyang Technological University, Singapore 639798, Singapore

**Keywords:** mesenchymal stem cells, exosomes, extracellular vesicles, cornea, scarring, wound healing, immunomodulation

## Abstract

Corneal scarring is a leading cause of worldwide blindness. Human mesenchymal stem cells (MSC) have been reported to promote corneal wound healing through secreted exosomes. This study investigated the wound healing and immunomodulatory effects of MSC-derived exosomes (MSC-exo) in corneal injury through an established rat model of corneal scarring. After induction of corneal scarring by irregular phototherapeutic keratectomy (irrPTK), MSC exosome preparations (MSC-exo) or PBS vehicle as controls were applied to the injured rat corneas for five days. The animals were assessed for corneal clarity using a validated slit-lamp haze grading score. Stromal haze intensity was quantified using in-vivo confocal microscopy imaging. Corneal vascularization, fibrosis, variations in macrophage phenotypes, and inflammatory cytokines were evaluated using immunohistochemistry techniques and enzyme-linked immunosorbent assays (ELISA) of the excised corneas. Compared to the PBS control group, MSC-exo treatment group had faster epithelial wound closure (0.041), lower corneal haze score (*p* = 0.002), and reduced haze intensity (*p* = 0.004) throughout the follow-up period. Attenuation of corneal vascularisation based on CD31 and LYVE-1 staining and reduced fibrosis as measured by fibronectin and collagen 3A1 staining was also observed in the MSC-exo group. MSC-exo treated corneas also displayed a regenerative immune phenotype characterized by a higher infiltration of CD163+, CD206+ M2 macrophages over CD80+, CD86+ M1 macrophages (*p* = 0.023), reduced levels of pro-inflammatory IL-1β, IL-8, and TNF-α, and increased levels of anti-inflammatory IL-10. In conclusion, topical MSC-exo could alleviate corneal insults by promoting wound closure and reducing scar development, possibly through anti-angiogenesis and immunomodulation towards a regenerative and anti-inflammatory phenotype.

## 1. Introduction

The cornea is the transparent part of the eye, essential in refracting light for normal vision. Corneal scarring, caused by various aetiologies such as infections, inflammation, trauma, and degenerative diseases [1,2,3,4,5,6,7,8], interrupts the normal passage of light into the eye, causing visual loss. Over 10 million people worldwide suffer from corneal blindness, among which 7% are due to corneal scarring [9]. Sufficient vision can be restored if the opacities in corneal scars are removed, or the density of the opacities is reduced. Visual impairment due to corneal scarring is one of the most common indications for corneal transplantations [10,11].

Although modern advanced techniques of lamellar keratoplasties can achieve high rates of success [7,8,12], there are fundamental limitations to conventional donor-reliant corneal transplantations. The replacement of scars from diseased corneas requires a supply of donor corneas. The donors must also be screened for transmissible diseases through robust serological testing. In addition, there has been a global restriction in corneal transplantations due to a worldwide shortage of suitable donor corneas [13]. Such problems with donor corneas availability have been further strained by the recent COVID-19 pandemic [14,15]. Furthermore, current corneal transplantation techniques rely on specialized surgical expertise, which is associated with high costs. There is also a prolonged post-operative visual rehabilitation process [8,10]. Inherent problems from allogenic transplants, such as long-term risks of immunological graft rejection and graft failure, also exist [8,16,17]. There is thus an impetus to search for alternative therapies for corneal diseases that are less dependent on donor availability [18].

Although the human cornea is known to be an immune-privileged tissue, immune-mediated inflammation can occur in the stroma following a significant insult, especially one that involves a disruption of the epithelium and Bowman’s membrane [19]. Such disruptions lead to the diffusion of growth factors and cytokines from the tear film into the exposed stroma, which in turn activates quiescent resident keratocytes [20]. The appearance of corneal stromal fibroblasts, a repair cell type transformed from keratocytes, is a key player in the local inflammatory response that is associated with stromal matrix remodeling and haze formation, disrupting light passage [20]. The fibroblasts release pro-inflammatory cytokines and chemokines, such as interleukin (IL)-6, IL-8, and tumor necrosis factor-α (TNF-α), that act as chemo-attractants to neutrophils and lymphocytes causing tissue inflammation [21]. In addition, they also synthesize complement components, regulating the complement cascade [22,23]. Complement activation triggered by pro-inflammatory stimuli, such as TNF-α or lipopolysaccharides, in turn, induces corneal inflammation via translocation of nuclear factor-kappa B (NFκB) [24,25]. Following stromal injury, resident innate immune cells in the cornea can also mount a rapid response [26]. For example, macrophages, depending on the biochemical signals, can become polarized toward pro-inflammatory M1 macrophages or reparative M2 macrophages [27]. Treatments that can modulate such inflammatory cascades can potentially limit the extent of downstream fibrosis and scar tissue formation and are thus promising therapeutic avenues to evaluate [15,28,29].

Mesenchymal stem cells (MSCs) are multipotent progenitors that originate from the mesoderm of adult tissues such as the bone marrow, blood, liver, spleen, adipose tissue, placenta, and umbilical cord blood [30]. MSCs are long known to have potent immunomodulatory activity and were first used to successfully treat severe acute graft-versus-host disease (GVHD) patients in 2004 [31]. This was followed by a successful multi-centered phase 2 trial in 2008 [32]. As a testament to the potent immunomodulatory activity of MSC, there are currently 745 trials (search terms: MSC|Immune System Disorder) registered at ClinicalTrials.gov to test MSC against a wide variety of immune diseases such as GVHD, diabetes, multiple sclerosis, systemic lupus erythematosus, and rheumatoid arthritis. Although such effects have been attributed to secreted soluble factors, including iNOS, IL-10, TGF-β, HGF, and PGE2, the role of these soluble factors cannot be unambiguously proven [33].

The mechanism of action of MSCs underwent a paradigm shift when it was reported that MSCs exert their effects through the paracrine activity of secreted nano-sized 80–1000 nm microvesicles or 100–150 nm exosomes [34,35]. Microvesicles and exosomes are collectively described as extracellular vesicles (EVs). Direct head-to-head comparisons between the secreted EVs and their parental cells confirmed that the EVs were the mediator of MSC activity as the therapeutic activity of the EVs and parental MSCs were comparable [34,36,37]. Furthermore, smaller MSC EVs of <160 nm but not >215 nm EVs exhibit protective effects [38], and small MSC-EVs of 50–200 nm, such as exosomes, are the most active EVs [39].

MSC exosomes (MSC-exo) are small EVs that carry a wide range of cellular molecules such as proteins, small nucleic acid fragments, lipids, and cytokines to mediate cell-cell communications [40]. Of the varied EV constituents, proteins are presently considered the main components [41]. Various studies have also reported the efficacy of MSC-exo in pre-clinical models of ocular diseases or injuries, such as experimental autoimmune uveitis [42,43], laser-induced retinal injuries [44], hyperglycemic-induced retinal inflammation [45], and choroidal neovascularization [46]. MSC-exo have also been shown to promote ocular repair and regeneration, such as retinal ganglion cell growth [47]. Such exosome-mediated effects could represent a non-cell-based (cell-free), therapeutic strategy.

It is now widely recognized that the cell source, culture, conditioning, and EV enrichment process, influence the identity and potency of MSC EV/exosome preparations [39,48]. To ensure reproducible and consistent performance, we have been using an immortalized monoclonal MSC line, cultured and conditioned under identical conditions for all our MSC-exo preparations [49]. These MSC-exo preparations have been shown to be non-tumorigenic [50] and therapeutic in various pre-clinical models of diseases such as myocardial ischemia, [35,51] liver injury [52], survival of allogeneic skin grafts [53], acute GVHD [54], cartilage injuries and osteoarthritis [28,55,56,57], aging [58], acute irradiation toxicity [59], and psoriasis [60]. More importantly, these preparations also reportedly possess many immune modulating attributes and properties that could mitigate the stromal insult-induced inflammation. For example, MSC-exo has been shown to inhibit the assembly of terminal C5b-9 complement complex through CD59, a known inhibitor of terminal complement activation complex formation and an abundant MSC exosomal protein [61]. This inhibition attenuates complement-mediated activation of neutrophils manifested as NETosis and IL-17 production. Exosomal EDA-fibronectin has also been reported to activate TLR4 and polarize anti-inflammatory M2-like monocytes, which is implicated in MSC exosome-mediated polarization of Tregs [53,54]. Likewise, exosomal CD73 activity has been implicated in MSC exosome-induced proliferation and migration of chondrocytes, and synthesis of collagen II and TGF-β1, after cartilage injury or osteoarthritis [28,56].

We hypothesized that MSC-exo can exhibit similar wound healing and immunomodulatory properties following corneal injuries. In this study, we evaluated if our MSC-exo preparation would attenuate corneal scarring, inflammation, and neovascularization in a rat model of anterior corneal stromal injury induced by irregular phototherapeutic keratectomy (irrPTK).

## 2. Results

### 2.1. In Vitro Cellular Uptake and Immunological Effects of Mesenchymal Stem Cells-Derived Exosomes

**Figure 1:**
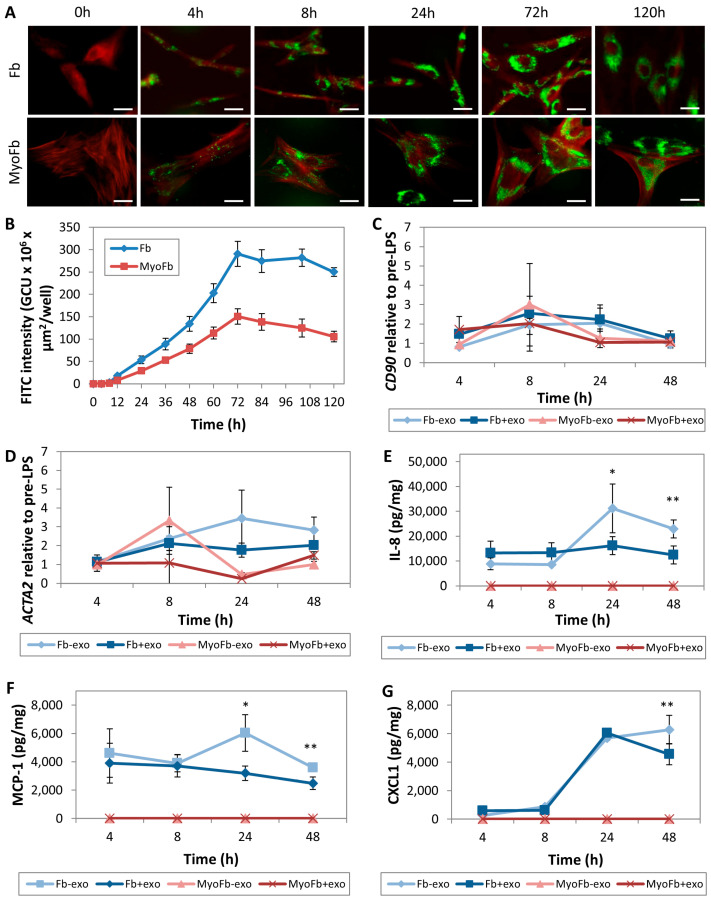
MSC-exo uptake by corneal stromal fibroblast (Fb) and myofibroblast (MyoFb) cultures and the cellular immunomodulatory effects. (**A**) Cellular uptake of AlexaFluor 488-tagged MSC-exo (green fluorescence) by cultured fibroblasts (CD90/Thy-1-positive; red fluorescence) and myofibroblasts (α-SMA-positive; red fluorescence). Scale bars: 50 µm. (**B**) The internalization of AlexaFluor 488-tagged MSC-exo was quantified by measuring the fluorescence intensity changes over 120 h using an IncuCyte live-cell system. (**C**,**D**) The immunomodulatory and anti-fibrotic effects of MSC-exo treatment on fibroblast and myofibroblast cultured were studied by target gene expression by RT-PCR. Expression fold change of *CD90* (**C**) and *ACTA2* (**D**) in fibroblast and myofibroblast cultures at 4, 8, 24, and 48 h after LPS treatments with or without MSC-exo. (**E**–**G**) The multiplex immunoassay showed the secretion of IL-8 (**E**), MCP-1 (**F**), and CXCL1 (**G**) from fibroblasts at 4, 8, 24, and 48 h treatments with (+exo) or without MSC-exo (-exo). Myofibroblast culture did not express any of the analyzed cytokines and chemokines. * *p* < 0.05; ** *p* < 0.001.

Corneal wound healing is initiated by the transition of surviving keratocytes to the repair-type fibroblasts and, at a later stage, into scar-associated myofibroblasts [62]. Although fibroblasts are not specialized immune cells, they trigger the recruitment of inflammatory cells through the secretion of chemokines and adhesion molecules [63]. Hence, we first studied the effect of MSC-exo on these two cell types. Using fluorescence microscopy and Incucyte live-cell imaging system, we observed that when fed with Alexa Fluor 488-labeled MSC-exo, CD90-positive fibroblasts, and α-SMA positive myofibroblasts were fluorescent at 4 h and reached maximum fluorescence at 72 h (Figure 1A,B).

Next, we investigated the effects of the MSC-exo on LPS-challenged fibroblasts and myofibroblasts. The LPS stimulation increased *CD90* and *ACTA2* expressions in fibroblasts and myofibroblasts (Figure 1C,D). Treatment with MSC-exo did not change the *CD90* expression in both cell types at 4, 8, 24, and 48 h (Figure 1C). Similarly, the MSC-exo did not alter *ACTA2* expression in both cell types at 4, 8, 24, and 48 h (Figure 1D).

We found that the MSC-exo treatment significantly reduced the secretion of IL-8 and MCP-1 from the fibroblasts at 24 and 48 h after LPS stimulation (Figure 1E,F), whereas CXCL1 from fibroblast was significantly suppressed at a later timepoint (48 h) in the presence of MSC-exo (Figure 1G). We did not detect the presence of IL-1β, IL-10, and TNF-α from the fibroblasts in any treatment group. The multiplex assay and ELISA did not detect IL-1β, IL-8, IL-10, MCP-1, CXCL1, and TNF-α secretion from the myofibroblasts, even in the presence of LPS.

### 2.2. Dosing and Retention of Topically Applied AlexaFluo-488-Labeled Exosomes on Corneal Stroma

**Figure 2:**
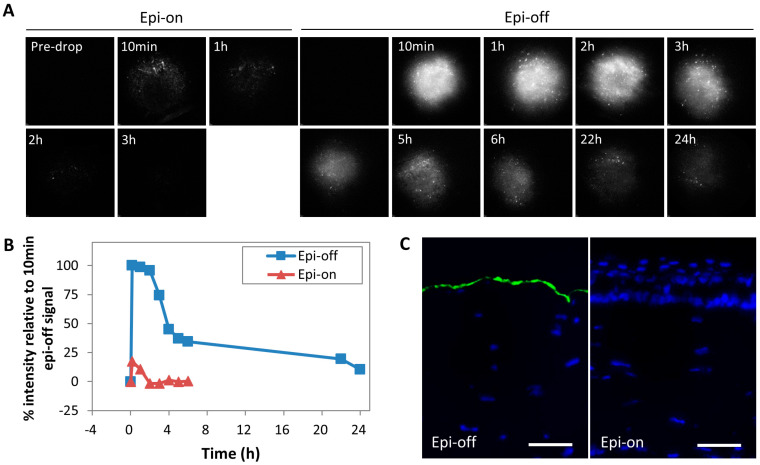
Time-lapse clearance of AlexaFluor 488-labeled MSc-exo to determine dosing of topical administration. (**A**) Fluorescent signals were detected using a confocal laser scanning ophthalmoscope. A rapid disappearance of signals (<1 h) was observed in epithelium-on corneas. Labeled MSC-exo applied to epithelium-off corneas showed signal retention for up to 24 h, with a time-dependent clearance of signals. (**B**) Signal intensity changes representing retention of AlexaFluor 488-labeled MSc-exo after topical application on rat corneas between epithelium-on and -off conditions (reference to peak intensity at 10 min on epithelium-off corneas). (**C**) Detection of labeled MSC-exo (green fluorescence) on epithelium-off corneal cryosections at 24 h. No fluorescent signal was detected from the epithelium-on corneas. DAPI (blue fluorescence) stained the nuclei. Scale bars: 100 µm.

A similar dose of AlexaFluor 488-labeled MSC-exo (4 µg protein in 8 µL volume) was applied to epithelium-on and -off corneas. At 10 min, the epithelium-on corneas retained <25% of the fluorescence on epithelium-off corneas, and the fluorescence was not detectable by 3 h (Figure 2A,B). In contrast, the epithelium-off corneas retained not only more fluorescence but also for a much longer time of 24 h (Figure 2A,B). Cryosections of epithelium-off corneas revealed that at 24 h, the fluorescence was restricted to the bare stromal surface. No fluorescent signal was seen for the epithelium-on corneas (Figure 2C). Together, this study demonstrated that the corneal epithelium forms an effective barrier against topically applied MSC-exo and prevents distribution of the exosomes into the corneal layer. Removal of the epithelium barriers allows topically applied MSC exosomes to enter and persist in the stroma for at least 24 h. However, the fluorescence was limited to the topmost layer of the stroma even after 24 h, suggesting the MSC-exo did not permeate the depth of the stroma and exit the stroma.

### 2.3. Effects of Mesenchymal Stem Cells-Derived Exosome Treatment on Epithelial Wound Closure

**Figure 3:**
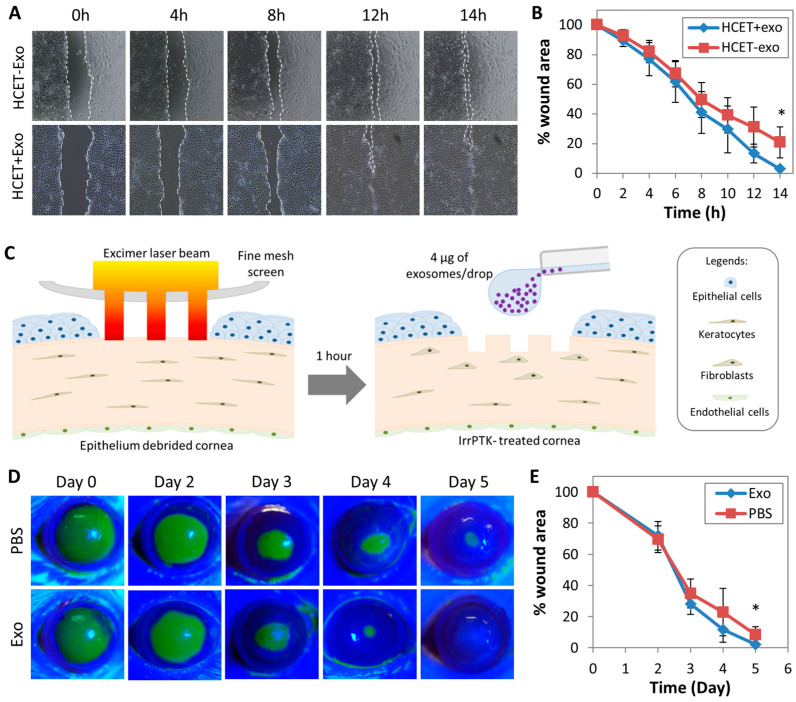
The effects of MSC-exo treatment on epithelial wound healing. (**A**) In vitro scratch wound assay on HCET culture showed that MSC-exo treatment induced a faster closure of denuded gap (upper panel) than the control group (lower panel). Photos were taken at 50× magnification. (**B**) Percentage changes of denuded gap area revealed a more rapid cell proliferation and migration into the wound area over 14 h by MSC-exo treatment. (**C**) Schematic illustrations of the corneal injury caused by irregular phototherapeutic keratectomy (irrPTK). Following the mechanical debridement of the corneal epithelium, excimer laser ablation was applied to the Bowman’s membrane and anterior stroma. Stromal irregularities were created by placing a fine screen mesh in the laser path. (**D**) Fluorescein staining of injured corneas revealed the different extent of epithelial wound closure after MSC-exo treatment versus PBS. (**E**) Percentage changes of wound area after MSC-exo versus PBS treatments. The wound closure was greater on day 5 after MSC-exo treatment. * *p* < 0.05.

The wound healing effects of MSC-exo were first assessed by an in-vitro scratch wound assay on HCET cell culture. The wound closed faster in the presence of MSC-exo treatment (Figure 3A), with a statistically significant difference at 14-h (3.0 ± 0.3% wound area in the MSC-exo treated group versus the control, 20.9 ± 10.4%) (*p* = 0.041) (Figure 3B). To confirm the in vitro results, we tested MSC-exo treatment in a rat model of acute corneal injury caused by irrPTK (Figure 3C). The corneal epithelial wound closed more rapidly after topical treatment with MSC-exo, compared to PBS-treated control corneas (Figure 3D). Statistically significant greater wound closure in MSC-exo group was observed on day 5 (2.3 ± 3.6% vs. 8.3 ± 5.3% wound areas; *p* = 0.045) (Figure 3E).

### 2.4. Effects of Topical Mesenchymal Stem Cells-Derived Exosome Treatment on Corneal Stromal Haze Development in Rat Corneas Injured by Irregular Phototherapeutic Keratectomy

**Figure 4:**
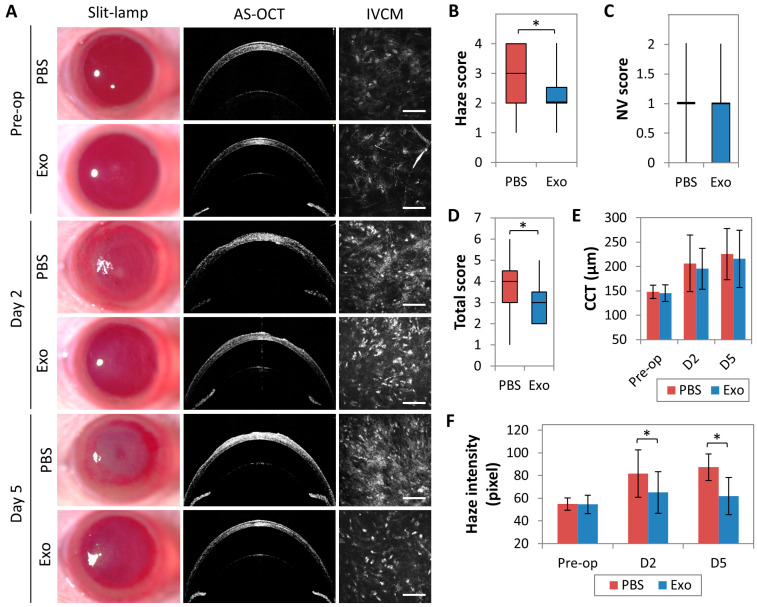
Evaluation of corneal stromal haze and corneal vascularization in eyes that had undergone irregular phototherapeutic keratectomy (irrPTK) with administration of topical mesenchymal stem cells-derived exosomes (MSC-exo) or phosphate-buffered saline (PBS). (**A**) Representative slit-lamp, anterior segment-optical coherence tomography (AS-OCT), and in vivo confocal microcopy images (IVCM) of rat corneas preoperatively and on days 2 (D2) and 5 (D5). Scale bars = 100 µm. Clinical grade scoring of corneas included a haze score (0–4, with 4 being the most severe opacification) (**B**), a neovascularization (NV) score (0–2, with 2 having vascularization >2 cm over the cornea) (**C**), and a total score (0–6, with 6 indicating the most severe opacification and neovascularization) (**D**). (**E**) Central corneal thickness (CCT) of the corneas preoperatively and at D2 and D5 postoperatively, measured from the AS-OCT images. (**F**) Comparative relative haze intensity at 10–15 µm-stromal depth quantified by stromal reflectivity using IVCM, showing a significantly lower haze intensity in the group receiving MSC-exo compared to the control. * *p* < 0.05.

After irrPTK injury, rat corneas topically treated with MSC-exo remained clear throughout the follow-up period, whereas PBS-treated control corneas showed progressive haze development (Figure 4A). On day 5 post-injury and treatment, the MSC-exo treated group had significantly lower haze score (median = 2; IQR = 0.5) than the PBS control (median = 3; IQR = 2) (*p* = 0.002; Figure 4B). There was no statistically significant difference in central corneal thickness (CCT), measured on AS-OCT, between both groups on day 2 (*p* = 0.588) and on day 5 (the end of examination) (*p* = 0.654) (Figure 4A,E). Stromal haze intensity, as measured with in vivo confocal microscopy, was reduced in MSC-exo treatment group from 65.2 ± 11.7 pixels on day 2 to 61.9 ± 16.3 pixels on day 5 but was increased in PBS control group from 81.7 ± 20.9 pixels on day 2 to 87.5 ± 18.3 pixels on day 5 (Figure 4F). On both day 2 and 5, the differences in stromal haze intensity between the treatment and control groups were statistically significant at *p* = 0.017 and *p* = 0.004, respectively (Figure 4F).

Neovascularization score in the MSC-exo treated group (median = 1; IQR = 1) was also lower than the PBS control group (median =1; IQR = 0) (*p* = 0.338; Figure 4C), but this was not statistically significant. Taking both the corneal haze and neovascularization scores into consideration, the corneas that received MSC-exo had substantially better clarity and were less vascularized (median total score = 3; IQR = 1.5) compared to the injured corneas receiving PBS only (median total score = 4; IQR = 1.5) (*p* = 0.004; Figure 4D).

### 2.5. Immunohistochemistry to Assess the Relative Molecular Changes in the Injured Corneas

**Figure 5:**
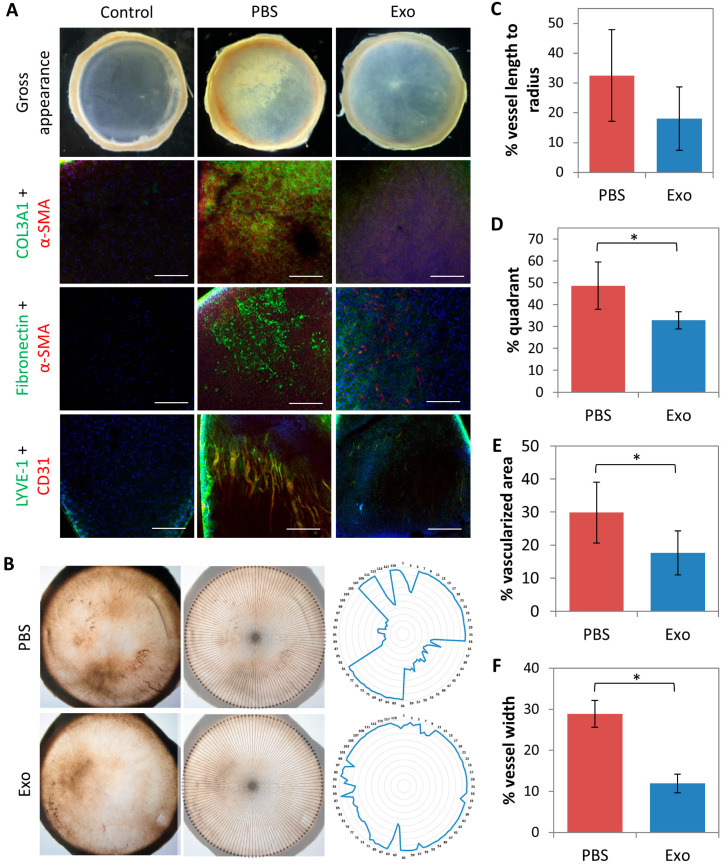
Gross appearance and immunohistochemistry studies of excised cornea. There was a reduction in the corneal haze in the mesenchymal stem cells-derived exosomes (MSC-exo) treated corneas compared to the phosphate-buffered saline (PBS) treated corneas under the stereomicroscope ((**A**); top panel). MSc-exo treated corneas had lower expression of fibronectin (green fluorescence), collagen 3A1 (green fluorescence), and α-SMA (red fluorescence) compared to PBS-treated corneas ((**A**); second and third row panels). CD31 (red fluorescence) with LYVE-1 (green fluorescence) colocalization staining on the excised corneas revealed a reduction of the vascularized area that received MSC-exo treatment compared to PBS ((**A**); bottom panel). DAPI (blue fluorescence) stained the nuclei. Quantification analysis showed that MSC-exo-treated corneas had reduced blood vessel lengths to the corneal radius (**B**,**C**), percentage quadrant with vessels (**B**,**D**), and percentage corneal area with vessel ingrowth (**B**,**E**), compared to PBS-treated corneas. Vessel width was significantly lower in the MSC-exo-treated corneas, compared to controls (**F**). * *p* < 0.05. Scale bars = 200 µm.

To identify the molecular changes in MSC-exo- and PBS-treated corneas after irrPTK, the corneas were isolated. Consistent with the above observations, corneas from the MSC-exo-treated group had visibly reduced haze compared to the PBS controls (Figure 5A; top panel). Immunohistochemistry revealed that PBS-treated control corneas but not MSC-exo-treated corneas were highly immunoreactive for corneal fibrosis markers, fibronectin, collagen 3A1, and α-SMA. The naive corneas were not immunoreactive for fibronectin, collagen 3A1, and α-SMA.

Consistent with the lower neovascularization score, MSC-exo-treated corneas had reduced CD31 and LYVE-1 immunoreactivity compared to PBS control corneas (Figure 5A bottom panel). MSC-exo treated corneas also had reduced vessel lengths to the corneal radius (18.0 ± 10.6% vs. 32.5 ± 15.4%; *p* = 0.126) (Figure 5B,C), significantly lower percentage quadrant with vessels (32.8 ± 3.9% vs. 48.7 ± 10.8%; *p* = 0.027) (Figure 5B,D), and less corneal area with vessel ingrowth (17.6 ± 6.6% vs. 29.8 ± 9.2%; *p* = 0.046) (Figure 5B,E). Width measurement of CD31 and LYVE1 immunoreactive structures were also significantly thinner vessel structures in the MSC-exo treated corneas, compared to controls (*p* < 0.0001) (Figure 5F).

### 2.6. In Vivo Immunomodulatory Effects of Mesenchymal Stem Cells-Derived Exosome Treatment

**Figure 6:**
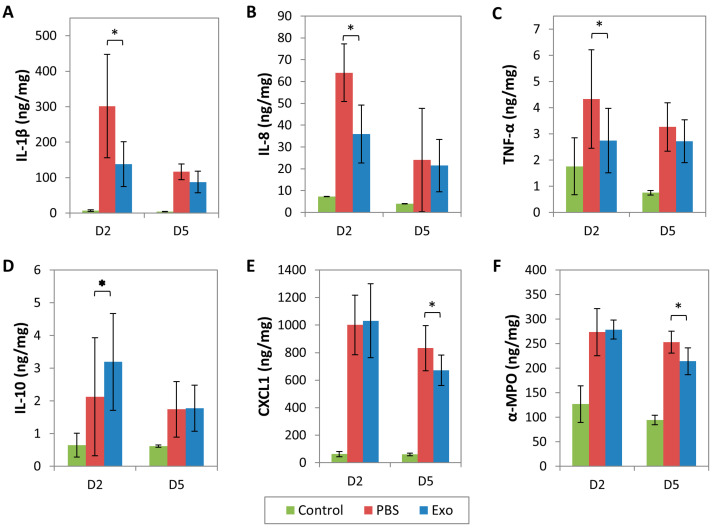
Host immune responses to laser-induced corneal scarring in rats on days 2 and 5 after topical application of mesenchymal stem cells-derived exosomes (MSC-exo) or phosphate-buffered saline (PBS). Pro-inflammatory cytokines IL-1β (**A**), IL-8 (**B**), and TNF-α (**C**) and anti-inflammatory IL-10 (**D**) expression were detected using multiplex immunoassay, whereas chemokine CXCL1 (**E**) and activated neutrophil marker α-myeloperoxidase (α-MPO) (**F**) expression were measured using ELISA. D2 = day 2; D5 = day 5. * *p* < 0.05.

Using Bio-Plex Multiplex Immunoassay, the tissue lysates of MSC-exo treated corneas treated statistically significant lower expression of pro-inflammatory cytokines, IL-1β, IL-8, and TNF-α (Figure 6A–C, Table S2) than PBS control group on day 2 but not day 5. Anti-inflammatory cytokine, IL-10, was statistically significantly higher in MSC-exo treated corneas relative to PBS control corneas on day 2 but not day 5 (Figure 6D, Table S2).

Unlike the other cytokines, a statistically significant difference in CXCL1 chemokine between the MSC-exo treated corneas and PBS control corneas was observed only on day 5 when CXCL1 was downregulated in the MSC-exo group (Figure 6E, Table S2). Likewise, α-MPO expression was also lower on day 5 in the MSC-exo group (Figure 6F, Table S2). Overall, the expressions of cytokines, chemokines, and neutrophils were significantly lower or negligible in the non-injured corneas (Figure 6A–F).

**Figure 7:**
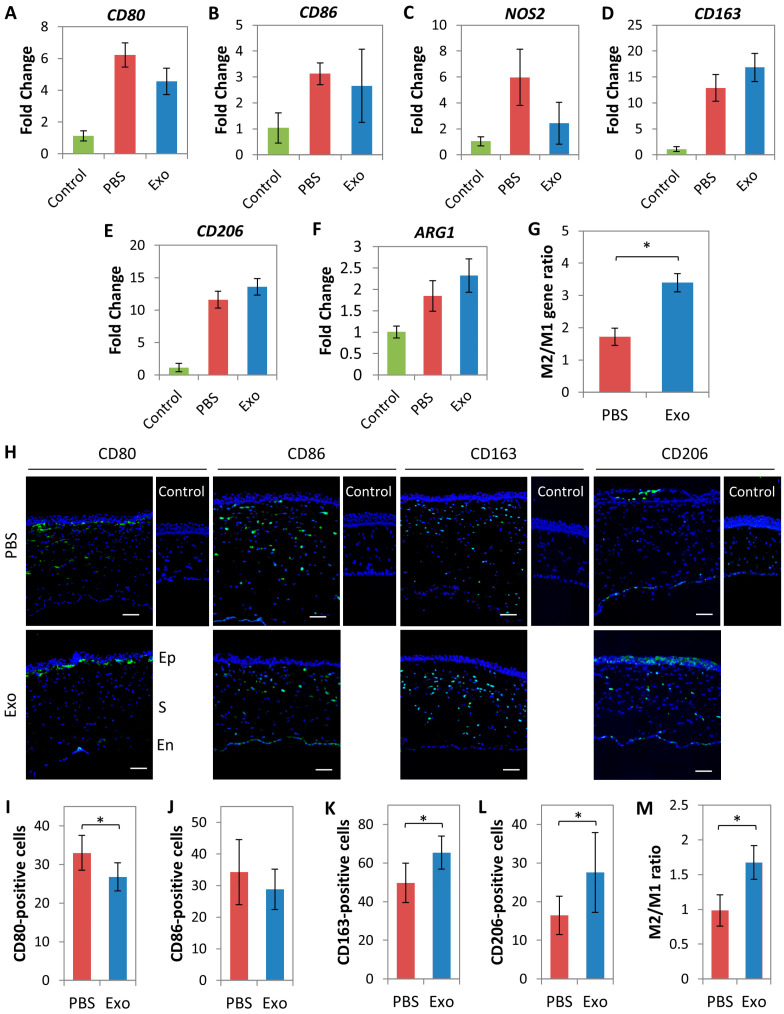
Macrophage polarization in laser-injured rat corneas after 5 days of MSC-exo or PBS topical application. Fold change of gene expression of M1 phenotype, *CD80* (**A**), *CD86* (**B**), and *NOS2* (**C**), and M2 phenotype, *CD163* (**D**), *CD206* (**E**), and *ARG1* (**F**) in the injured corneas from the uninjured corneas. (**G**) M2/M1 gene ratio was significantly higher in the corneas treated with MSC-exo. (**H**) Corresponding immunohistochemistry of M1 and M2 polarized macrophage markers was performed and viewed at the laser-injury site. The images were orientated such that the corneal epithelium (Ep) appeared at the top, followed by the stroma (S), and the endothelium (En) at the bottom. The cells expressing M1 polarized macrophage markers, CD80 (**I**) and CD86 (**J**), were counted and compared to those expressing M2 macrophage markers, CD163 (**K**) and CD206 (**L**). (**M**) The M2/M1 positive-cells ratio was significantly higher in the corneas treated with MSC-exo than in the PBS group. * *p* < 0.05. Scale bars = 50 µm.

The anti-inflammatory responses after topical MSC-exo treatment were also demonstrated by the downregulated expression of pro-inflammatory M1 macrophage-associated genes (*CD80*, *CD86*, and *NOS2*) on day 5, when compared to PBS control group (Figure 7A–C). In contrast, the anti-inflammatory M2 macrophage-associated genes (*CD163, CD206*, and *ARG1)* were strongly expressed in corneas treated with MSC-exo (Figure 7D–F). Individually, the differences in the M1 and M2 polarized macrophage gene expressions were not significantly different between MSC-exo and PBS groups. However, collectively, the M2/M1 gene ratio was significantly higher in the MSC-exo treated group compared to the PBS control group (3.4 ± 0.3 vs. 1.7 ± 0.3; *p* = 0.002) (Figure 7G).

The RNA expression results were supported by the immunostaining experiments of M1 and M2 macrophage markers on corneal sections. In Figure 7H, the quantitative results showed a lower number of CD80-positive cells (27 ± 4 cells vs. 33 ± 5 cells; *p* = 0.006) (Figure 7I) and CD86-positive cells (29 ± 6 cells vs. 34 ± 10 cells; *p* = 0.201) (Figure 7J) and higher number of CD163-positive cells (65 ± 9 cells vs. 50 ± 10 cells; *p* = 0.003) (Figure 7K) and CD206-positive cells (28 ± 10 cells vs. 16 ± 5 cells; *p* = 0.013) (Figure 7L) in the MSC-exo treated corneas compared to controls. Collectively, the M2/M1 ratio was significantly higher in the corneas treated with MSC-exo (1.7 ± 0.2 vs. 1.0 ± 0.2; *p* = 0.023) (Figure 7M). Naive corneas had a negligible expression of any of these markers (Figure 7H).

## 3. Discussion

Our study demonstrated that human MSC exosomes are therapeutically efficacious against corneal injury in both in vitro and in vivo models. Specifically, we showed that topically applied MSC-exo in an excimer laser-induced rat corneal injury model improved the rate of corneal epithelial wound healing, reduced corneal haze development, and suppressed corneal neovascularization. These observations were consistent with reduced immunoreactivity for molecular markers specific for corneal fibrosis and angiogenesis in the MSC-exo treated corneas, namely, fibronectin, collagen 3A1, α-SMA, CD31, and LYVE-1. As inflammation is a major driver of fibrosis and neovascularization, we observed that there was a downregulation of pro-inflammatory M1 macrophage-associated genes with corresponding upregulation of the anti-inflammatory M2 markers in MSC-exo treated corneas. In addition, the level of pro-inflammatory cytokines IL-1β, IL-8, and TNF-α in the MSC-exo-treated corneal tissue lysate was reduced with a concomitant increase in anti-inflammatory IL-10. Overall, our results demonstrate that topically applied MSC-exo alleviates corneal insults by promoting wound healing and preventing scar development through the modulation of injury-induced inflammation towards a regenerative immune phenotype.

In our animal study, we referenced the dosage of MSC-exo for topical application on rat corneas to an earlier study where topically applied exosomes were used to reduce psoriatic inflammation [60]. The dosage was a QD topical application of 16 µg protein/cm^2^. For rat corneas with 5.5 mm diameter, an equivalent QD dosage would be 4 µg protein in 8 µL to cover the entire surface. To assess the biodistribution of topically applied exosomes on corneas, fluorescent exosomes were applied on intact corneas (“epithelium on”) or injured corneas (‘epithelium-off’). In ‘epithelium-on’ corneas, the fluorescent signals of MSC-exo were cleared within an hour, indicating that topical application of MSC-exo on normal corneas with rapid tear fluid turnover corneal surface will not persist or permeate the stroma. In the ‘epithelium-off’ corneas, the fluorescence permeated the stroma and decayed slowly over 24 h. As 50% fluorescence remained after 3 h, topical MSC-exo treatment was applied at 3 hourly dosing intervals for 5 days in our study. This is to ensure MSC-exo bioavailability in the corneal stromal during the acute phase of corneal injuries prior to epithelial wound closure, which usually occurs 4–5 days following corneal injuries.

When AlexaFluor 488-labeled MSC-exo was applied to cell cultures, we also observed that MSC-exo was uptaken by cultured fibroblasts and myofibroblasts, and the intracellular detection of fluorescent signals were apparent from 4 h to over 120 h, with intracellular signals peaking at 72 h. The MSC-exo treatment did not alter fibroblast phenotypes but exerted a direct immunomodulatory effect by modulating the expression and secretion of chemo-attractants, IL-8, MCP-1, and CXCL1 at around 24 to 48 h after insult. Interestingly, MSC-exo did not affect the secretion of cytokines or chemo-attractants from cultured myofibroblasts. Taking the aforementioned findings into consideration, MSC-exo therapies may thus be most beneficial clinically when applied in the acute phase of corneal insults prior to epithelium healing to modulate the production of chemo-attractants by the stromal fibroblasts, an early event committing to fibrosis development and neovascularization, prior to late myofibroblast transdifferentiation and scar formation. This also formed the basis of our in vivo study using an acute corneal haze model. It must be pointed out that in addition to corneal fibroblasts, it has been observed that myofibroblasts can also develop from other progenitors such as bone marrow-derived cells (fibrocytes) [64], Schwann cells [65], and epithelial cells [66]. Nevertheless, understanding the roles of these latter progenitors in corneal scar formation is still largely unknown, and it was beyond the scope of our study to evaluate the effects of MSC-exo on these cell types.

A key finding in this study is the dominant effect of MSC-exo in modulating the immune phenotype during a corneal injury in vivo. The injured corneas treated with MSC-exo exhibited significantly lower expressions of pro-inflammatory cytokines, IL-8 on day 2 and CXCL1 on day 5. Interestingly, the downregulated CXCL1 on day 5 may have attenuated a neutrophil influx, indicated by a correspondingly lower level of α-MPO expression in the MSC-exo treated corneas. In addition, the reduced expressions of IL-1β and TNF-α, on day 2 may have also played a role in modulating corneal fibrosis and neovascularization [67]. Furthermore, we showed that MSC-exo may enhance wound healing through immunomodulation as evidenced by (1) a macrophage polarization towards regenerative M2 macrophages with an observed higher M2/M1 macrophage-associated gene expression ratio and amplified presence of M2 over M1 macrophages, (2) reduced levels of M1 macrophage-associated pro-inflammatory cytokines, IL-1β and TNF-α, and (3) increased levels of anti-inflammatory cytokine, IL-10, in MSC-exo treated corneas compared to PBS-treated controls. The capacity of MSC exosomes to polarize macrophages toward a M2 phenotype was first reported in 2014 [53]. This effect of tissue repair and regeneration has since been implicated in animal models of osteochondral defects and osteoarthritis [28,29].

The attenuation of corneal stromal inflammation with MSC-exo treatment may have also resulted in the observed enhancement of corneal epithelial wound closure. Corneal injuries that involve the disruption of the epithelium and Bowman’s membrane trigger stromal inflammatory cascades due to the permeation of cytokines and chemokines (e.g., IL-1, IGF-1, and TGF-β1) from the corneal epithelium and tear film into the stroma [67]. These factors are thought to activate fibroblast activity to recruit inflammatory cells into the stromal tissue and myofibroblasts to produce abnormal ECM, resulting in corneal opacities. The earlier closure of epithelial wound after MSC-exo treatment might reduce such ‘cytokine and chemokine floodgates’, thereby further modulating the corneal inflammation and haze formation. Such enhancement of epithelial wound closure, promoted by exosomes derived from corneal epithelial cells and corneal MSCs, has also been observed by other investigators [68,69].

Although designed to limit tissue injury and promote repair, the inflammatory responses of any injured tissue can be a double-edged sword, as excessive and persistent inflammation damages healthy neighboring tissue [70]. Furthermore, scarring is the pathological end-stage complication of inflammation. At present, the mainstay of treatment of inflammation is non-specific immunosuppressive medications (e.g., corticosteroids). However, such medications are associated with significant adverse effects, which include secondary infections as a result of non-specific suppression of host immune defenses. This is important in cases of corneal infections, especially in fungal keratitis, where corticosteroids are often not administered at the outset due to the risks of non-specific immunosuppression and worsening infections. Such planned delays in starting corticosteroids, however, may result in uncontrolled inflammation and ultimately, fibrosis. By immunomodulation and effecting a regenerative phenotype within the injured tissues, MSC-exo could potentially be a more effective alternative therapy for corneal insults compared to traditional immunosuppressive therapies.

There are limitations to this study which the authors acknowledge. In our experiments where we evaluated the signal retention of labeled exosomes, applied to ‘epithelium on’ versus ‘epithelium off’ naive corneas, we were working on the assumption that the emitted signals were from functional intact exosomes. We felt that signal retention was a good starting point for the determination of the dosing frequency of this novel therapy for the cornea. We also assumed that the continuous exosome exposure would maximize the potential of the MSC-exo within the relatively short but most effective therapeutic window (4–5 days before complete epithelial closure). In our in vitro experiments, we observed internalization of exosomes in the fibroblasts and myofibroblasts. Once again, this worked on the assumption that the uptake of exosomes into the target cells was important in inducing their therapeutic effects. However, we cannot rule out that the MSC-exo could also affect target cells extracellularly and sometimes even indirectly through mediator cells. Despite these assumptions, the wound-healing effects of the MSC-exo using the inferred dosing were demonstrated in our subsequent experiments. Nevertheless, we acknowledge that the optimal dosing and potentially more effective exosome delivery routes to the corneal stroma require further investigation.

## 4. Material and Methods

### 4.1. Purified Exosomes from Embryonic Stem Cell-Derived Mesenchymal Stem Cells

MSC-exo fractions were prepared as described previously [35,51]. Briefly, a monoclonal immortalized MSC line, E1-MYC 16.3 was cultured in DMEM with 10% fetal calf serum to 80% confluency as previously described [49]. The cells were washed with PBS and cultured in a chemically defined medium for three days to generate a conditioned medium (CM) as previously described [35,71,72]. The chemically defined medium was prepared as follows: 480 mL DMEM (Thermo Fisher, Waltham, MA, USA, #31053), 5 mL NEAA (Thermo Fisher, #11140-050), 5 mL L Glutamine (Thermo Fisher, #25030-081), 5 mL Sodium Pyruvate (Thermo Fisher, #11360), 5 mL ITS-X (Thermo Fisher, #51500-056), and 0.5 mL 2-ME (Thermo Fisher, #21985-02). This was supplemented with 0.1 mL bFGF (0.5 ng/µL 0.2%BSA in PBS (+) and 0.005 mL PDGF (100 ng/µL PBS (+)). These latter components were obtained as follows: Bovine Serum Albumin or BSA (Sigma-Aldrich, St. Louis, MO, USA, #A9647), PDGF (AB CYTOLAB, #100-00), bFGF (Thermo Fisher, #13256-029), and PBS (+) (Thermo Fisher, #14040-133). The CM was size fractionated and concentrated 50× by tangential flow filtration using a membrane with a molecular weight cut-off (MWCO) of 100 kDa (Sartorius, Goettingen, Germany, #VS15T41) to generate the MSC exosome preparation. The MSC exosome preparation was characterized in accordance with recommended metrics for MSC-small EVs [39]. It was assayed for protein concentration using a Coomassie Plus (Bradford) Assay Kit (Thermo Fisher, #23236), and the exosome preparations were quantified by the protein concentration. All batches of exosome used in this study were determined by Nanoparticle tracking analysis on a ZetaView instrument (Particle Matrix GmbH, Ammersee, Germany) to have 1.46 × 10^11^ ± 2.43 × 10^10^ particles per μg protein and particle modal size of 138.62 ± 4.45 nm using the parameters (sensitivity = 90, shutter = 70, frame rate = 30, min brightness = 25, min area = 5, max area = 1000). Each batch of MSC exosome preparation was also confirmed by Western or ELISA to have CD81 and CD73. The exosome preparations were filtered with a 0.22 µm filter (Merck Millipore, Billerica, MA, USA) and stored in −20 °C freezer until they were used.

### 4.2. Fluorescence Labeling of Exosomes

Exosomes were labeled by incubating 1 mg exosomes in 0.8 mL PBS with 1 mg AlexaFluor 488 amine-reactive probe (Thermo Fisher) in a final 1 mL volume of 0.1 M sodium bicarbonate buffer (Sigma-Aldrich) with gentle agitation and protection from light for 1 h at room temperature (~25 °C). Excess unreacted probes were removed by passing the mixture through Bio-Gel P30 gel columns (#7326231, Bio-Rad Laboratories, Hercules, CA, USA). The labeled exosomes in the flow-through were sterile filtered with 0.22 μm filters (Merck Millipore).

### 4.3. Primary Corneal Stromal Fibroblast and Myofibroblast Culture

Research grade cadaveric human corneal tissues procured from Lions Eye Institute for Transplant and Research (Tampa, FL, USA) were transported in Optisol-GS (Bausch and Lomb, Irvine, CA, USA) at 4 **°**C and cultured immediately on arrival. The central button was trephined, followed by gentle scraping to remove the corneal epithelium and endothelium. The corneal stroma was digested with collagenase I (1 mg/mL; Worthington Biochemical Corp., Lakewood, NJ, USA) in DMEM/F12 (Thermo Fisher) overnight at 37 **°**C. Isolated cells were harvested and cultured in DMEM/F-12 containing fetal bovine serum (FBS; 10%; Thermo Fisher) and 1% penicillin and streptomycin sulfate (Thermo Fisher) to generate stromal fibroblasts. To generate myofibroblasts, fibroblasts at passages 3 to 6 were cultured in the presence of recombinant human TGF-β1 (1 ng/mL; R&D Systems, Minneapolis, MN, USA) for 3 days.

### 4.4. In Vitro Cellular Uptake of Exosomes

Corneal fibroblasts (*n* = 4) and myofibroblasts (*n* = 4) were seeded at a density of 3 × 10^3^ cells/cm^2^ in a 96-well plate in serum-free DMEM/F-12 media (SFM) overnight. AlexaFluor 488-tagged MSC-exo (4 μg protein) was added and incubated in an IncuCyte ZOOM imaging system (Essen BioScience, Ann Arbor, MI, USA) for 120 h. At 0, 4, 8, 24, 72, and 120 h, the cells were stained with mouse monoclonal anti-CD90/Thy-1 (BD Biosciences, Franklin Lakes, NJ, USA) and α-smooth muscle actin (α-SMA; Agilent, Santa Clara, CA, USA) antibodies, respectively, followed by goat anti-mouse Red-X conjugated IgG secondary antibody (Jackson ImmunoResearch, Wet Grove, PA, USA) to contrast the internalized AlexaFluor 488-tagged MSC-exo.

### 4.5. In Vitro Inflammatory Assay by Lipopolysaccharide Treatment

Cultures of fibroblasts (*n* = 3) and myofibroblasts (*n* = 3) seeded at a density of 10^4^ cells/cm^2^ in a 48-well plate were incubated in SFM containing lipopolysaccharide (LPS; 100 ng/mL; Sigma-Aldrich) for 24 h, as previously described [73]. After washes, the cells were either treated with MSC-exo (4 μg protein/mL) or PBS for 120 h. At 0, 4, 8, 24, 48, and 120 h, the conditioned media were collected for enzyme-linked immunosorbent assays (ELISA) or multiplex assay to detect inflammatory marker expression, and the cells were harvested for RNA analysis.

### 4.6. Corneal Epithelial Scratch Wound Assay

Human SV-40 immortalized corneal epithelial cell line HCET (RCB1384, Riken Cell Bank, Ibaraki, Japan) were seeded in 24-well culture plates and grown to confluency in DMEM/F-12, supplemented with 5% FBS [74]. Scratches were made with a P200 pipette tip. After washes, the cells were either treated with MSC-exo (4 μg protein/mL; *n* = 4) or with PBS (*n* = 4). Every 2 h, for a total of 14 h, the denuded area at fixed positions was captured using a Carl Zeiss Axioplan 2 microscope (Carl Zeiss, Oberkochen, Germany), and the area was quantified with ImageJ software version 1.54a.

### 4.7. Time-Lapse Tracing Studies: Dosing and Retention of AlexaFluo-488-Labeled Exosomes on Corneal Stroma

All animal experimentation followed the guidelines of the Use of Animals in Ophthalmic and Vision Research, The Association for Research in Vision and Ophthalmology (ARVO) Statement and was approved by the Institutional Animal Care and Use Committee (IACUC) of SingHealth, Singapore (protocol 2018/SHS/1446). To determine the optimal dosing frequency by topically administration, the retention of MSC-exo within normal corneas of Sprague–Dawley rats (6 to 8 weeks old) were examined in both intact (corneal epithelium-on, *n* = 5) and de-epithelialized (epithelium-off, *n* = 5) conditions. Rats were anesthetized by intraperitoneal ketamine hydrochloride (80 mg/kg; Parnell Lab., Alexandria, Australia) and xylazine (12 mg/kg; Troy Lab., Glendenning, Australia). The corneal epithelium was wetted with 5% ethanol for 10 s, followed by saline rinses and scraping using a surgical blade (#15; BD Pharmingen, Franklin Lakes, NJ, USA) sparing the limbus. AlexaFluor 488-labeled MSC-exo (4 µg protein in 8 µL volume) was topically applied at time 0. Corneas were examined using a confocal laser scanning ophthalmoscope (Spectralis, Heidelberg Engineering GmbH, Heidelberg, Germany) with a lens of 30**°** field of view and an excitation filter under fluorescein mode and intensity setting to 90. Time-lapse corneal pictures with labeled exosomes were recorded at time 0, and every 10 min for the first hour, then hourly for the next six hours, and then every six hours until the fluorescent signals disappeared. At least five in-focused images were chosen at each time point for Quantity One 1-D Analysis (Bio-Rad). The grey-scale images were imported, and signal area and density were measured. After background subtraction (images before treatment), the percentages of fluorescence intensity were calculated with reference to the peak intensity reading at the time of 10 min (after topical exosomes). Following rat euthanization at 24 h after topical application, the corneas were dissected and embedded in an optimal cutting temperature (OCT) compound. Serial cross-sections with a thickness of 6 µm were viewed under a Zeiss Axioplan 2 fluorescence microscope (Carl Zeiss) to detect AlexaFluor 488-tagged MSC-exo in the epithelium-on and epithelium-off corneas.

### 4.8. Rat Corneal Opacity Model by Irregular Phototherapeutic Keratectomy (irrPTK) and Exosome Eyedrops

Sprague–Dawley rats (6 to 8 weeks old, *n* = 76) were treated under general anaesthesia. The surgeries were performed by HSO and JSM. Only one eye of each rat was used for the experiments. The rat model of corneal opacity was created as previously described [75]. The breakdown of the number of rats used in each experiment was tabulated in Table S1. The eyes first received topical analgesic, lignocaine hydrochloride (1%; Pfizer, Brooklyn, NY, USA). The corneal epithelium was removed by ethanol treatment and scraping sparing the limbus. Anterior central stroma was ablated by irrPTK using a Technolas 217z excimer laser (Bausch and Lomb, Rochester, NY, USA) with the settings of a 3 mm ablation zone and an ablation depth of 15 µm. IrrPTK was performed by placing a fine mesh screen in the path of the laser after firing 50% of the pulses to induce stromal irregularity in the corneal stroma. After saline rinsing for 10 s, the corneas received topical tobramycin (1%; Alcon, Geneva, Switzerland).

MSC-exo were topically applied to the mouse corneas one hour after irrPTK. Lyophilized MSC-exo were reconstituted at a concentration of 0.5 µg protein/µL with injection water. A volume of 8 µL (representing 4 μg protein) was applied on the injured corneal surface every 3 hourly (determined by a half-life study as described below), 6 times a day for 5 days until the corneal epithelium healed. PBS drops (8 μL volume) were administered to the injured corneas of the control group.

### 4.9. Ophthalmic Examinations and Measurements

Rat corneas were examined 3 days prior to corneal injury or treatment to obtain the naïve reference and on days 2 and 5 postoperatively. Corneal changes were assessed using a Zoom Slit Lamp NS-2D (Righton, Tokyo, Japan). The manifestation of corneal haze and neovascularization at postoperative day 5 was graded according to the modified Hackett-McDonald cornea scoring system [76]. Corneal re-epithelialization was assessed with cobalt blue light of the slit-lamp apparatus after instillation of 2% sodium fluorescein (Bausch and Lomb). Corneal cross-section visualization was done using an RTVue anterior segment-optical coherence tomographer (AS-OCT; Optovue Inc., Fremont, CA, USA) and the central corneal thickness (CCT) was measured as a mean of 3 measurements taken at the center and at 0.5 mm on either side, respectively [77]. Optical sections along the corneal depth were obtained by in vivo confocal microscopy using Heidelberg retinal tomography HRT3 with Rostock corneal module (Heidelberg Engineering GmbH, Germany). All corneas were examined centrally with at least 3 *z*-axis scans from the corneal epithelium to endothelium. Semi-quantitative analysis of stromal reflectivity at 10–15 µm depth was performed using ImageJ (National Institute of Health, Bethesda, MD, USA) after the images were tonal-adjusted to map the actual pixel values using PhotoShop CC (Adobe Systems Inc., San Jose, CA, USA). Rats were sacrificed by overdosed intraperitoneal pentobarbital (Jurox, Rutherford, Australia). Both eyes were enucleated on day 2 (*n* = 18) and 5 (*n* = 58). The isolated corneas were imaged under a stereomicroscope with indirect illumination and processed for immunohistochemistry, ELISA, multiplex immunoassay, or RT-PCR.

### 4.10. Assay of Corneal Neovascularisation

Isolated mouse corneas were fixed in 4% paraformaldehyde (Sigma-Aldrich) for 1 h and washed with PBS. The whole corneas were blocked with 5% normal goat serum (NGS; Thermo Fisher) and 2% BSA and permeabilized with 0.15% saponin (Sigma-Aldrich) for 30 min at room temperature, then incubated with mouse monoclonal anti-CD31 antibody (Thermo Fisher) for 2 h. After washes, the samples were placed in secondary IgG-horseradish peroxidase conjugate antibody (Jackson ImmunoResearch) for one hour. The samples were washed, and signals were revealed by 3,3′-diaminobenzidine (DAB; Sigma-Aldrich) reduction and examined under brightfield microscopy (Carl Zeiss Axioplan 2). Whole mount pictures (*n* = 5 in each group) were obtained, and the proportion of vessel ingrowth from limbal vasculature was assessed by overlaying a radial plot of 120 lines (giving 3° separation slot). The length of vessel ingrowth along each line was recorded and presented as a radial chart. The changes in the vascularized area were compared between treatment and control groups. Separately, wholemount images after double immunostaining for CD31 and LYVE-1 (protocol in the following section) were imported to ImageJ, and the width of CD31+ and LYVE+ vessels was recorded at multiple sites (*n* = 50 for each cornea and total *n* = 5 corneas in each treatment group). Mean vessel widths were calculated.

### 4.11. Immunohistochemistry

Rat corneas were fixed in freshly prepared neutral buffered 2% paraformaldehyde. After PBS rinses, they were cut into quadrants for wholemount immunostaining (*n* = 3 corneas in each group). Another 3 corneas in each group were embedded in the OCT compound for cryosectioning at a thickness of 6 µm. Samples were treated with ice-cold 50 mM ammonium chloride (Sigma-Aldrich), saponin-permeabilized, and blocked with 2% BSA and 5% NGS, followed by incubation with primary antibodies: Mouse monoclonal anti-fibronectin (Sigma-Aldrich), CD31 (Thermo Fisher), CD80 (Lifespan Biosciences, Seattle, WA, USA), CD163 (Bio-Rad Laboratories) and α-SMA (Agilent) antibodies, and rabbit polyclonal anti-collagen 3A1 antibody (Novus Biologicals, Littleton, CO, USA), LYVE-1 (Abcam, Cambridge, UK), CD86 (Abcam) and CD206 (Abcam) antibodies for 2 h at room temperature. After PBS washes, the signals were revealed with appropriate Red-X– or AlexaFluor 488–conjugated IgG secondary antibody (Jackson ImmunoResearch) for 1 h at room temperature, washed and mounted with Fluoroshield with DAPI (4,6-diamidino-2-phenylindole; Santa Cruz Biotech, Santa Cruz, CA, USA). Samples were viewed under fluorescence microscopy (Carl Zeiss Axioplan 2). Cells expressing CD80-, CD86-, CD163- or CD206 were quantified from 3 random viewing fields of the central cornea. The M2/M1 ratio was calculated as follows:

M2/M1 ratio = ∑(CD163 + CD206 positive cells)/∑(CD80 + CD86 positive cells)(1)

Serial optical z-stack images (1 um thickness) on wholemount corneas were obtained for fibronectin, collagen 3A1, α-SMA, CD31, and LYVE-1 staining by laser-scanning confocal microscopy (TCP SP8, Leica, Wetzlar, Germany).

### 4.12. Gene Expression by Real-Time Polymerase Chain Reaction

Human fibroblasts, myofibroblasts, and HCET cells (*n* = 3 cultures in each treatment group) and rat corneal tissues (*n* = 9 in each group) were collected in TRIzol (Thermo Fisher) and extracted with chloroform-isoamyl alcohol (Sigma-Aldrich) followed by RNeasy kit (Qiagen, Valencia, CA, USA) and on-column RNase-free DNase kit (Qiagen) according to the manufacturer’s protocol. Three rat corneas were pooled to generate 1 biological sample. Reverse transcription of total RNA (1 μg) was performed with a Superscript III RT-PCR kit (Thermo Fisher). Gene expression was assayed with specific primer pairs (Table 1) by RT-PCR using Sybr Green Supermix (Bio-Rad) for human samples or TaqMan Gene Expression Assays (Applied Biosystems, Carlsbad, CA, USA) for rat samples. Experiments were run in triplicates. Relative gene expression levels of each sample (DCT) were normalized by the mean CT value to either housekeeping human glyceraldehyde-3-phosphate dehydrogenase (*GAPDH*) or rat hypoxanthine guanine phosphoriboxyl transferase (*HPRT1*). Macrophage polarization M1/M2 ratio was used to evaluate the ratio of gene expression of rat M1 (*CD80*, *CD86,* and *NOS2*) and M2 macrophage markers (*CD163*, *CD206,* and *ARG1*). M2/M1 ratio is defined as follows:

M2/M1 ratio = ∑(M2 marker fold change/*HPRT1*)/∑(M1 marker fold change/*HPRT1*) (2)

### 4.13. Enzyme-Linked Immunosorbent Assay and Multiplex Immunoassay

Rat corneas (*n* = 9 in each treatment group) without residual iris and scleral tissues were trimmed into small pieces and placed in 300 ul tissue lysis buffer (Thermo Fisher) containing protease inhibitor cocktail (Complete^TM^; Roche, Basel, Switzerland) and 1 mM phenylmethyl sulfonylfluoride (PMSF; Sigma-Aldrich) and disrupted by sonication with 4 times 30-s bursts on ice. Three rat corneas were pooled to generate 1 biological sample. The lysate was centrifuged at 14,000× *g* for 15 min at 4 **°**C. The supernatant was collected for the expression assay of CXCL1 and α-myeloperoxidase (α-MPO) was determined using a fluorometric immunoassay (R&D Systems) following the manufacturer’s instructions. The expression of IL-1β, IL-8, IL-10, MCP-1, and TNF-α was analyzed using Bio-Plex Multiplex Immunoassay (Bio-Rad). Three rat corneas were pooled to generate one biological sample. The samples were analyzed in triplicates, and the expression values were determined from standard curves. Separately, the conditioned media from fibroblast and myofibroblast cultures, challenged with LPS or not, were subjected to ELISA and multiplex immunoassay of the same markers (human-specific).

### 4.14. Statistical Analyses

Data were managed in Excel (Microsoft, Redmond, WA, USA) and analyzed using Statistical Program for Social Sciences (SPSS) Version 23 (IBM, Armonk, NY, USA). Differences in the distribution of continuous variables between groups were analyzed using the non-parametric Mann–Whitney U test and one-way ANOVA. The intra-class correlation coefficient (ICC) was used to evaluate inter-observer agreement levels of stromal haze grading. The significance level was set at *p* < 0.05

## 5. Conclusions

This pre-clinical study provides a scientific rationale for the use of MSC-exo to promote healing and prevent scarring in corneal injuries. This approach of using exosomes has the additional advantage of being acellular, negating the potential risks of allogenic tissue transplantations. From a translational perspective, the use of MSC-exo has the benefits of being standardized when manufactured and, compared to the use of other cell-based therapies, safer as they are immunologically inert and stable.

## Figures and Tables

**Table 1:** Primer sequence used in the RT-PCR analysis.

Gene	Accession No.	Sequence/Assay ID
*CD90* (human)	NM_006288	F: GACCCGTGAGACAAAGAAGCR: TGGAGTGCACACGTGTAGG
*ACTA2* (human)	BC017554	F: CCTCCCTTGAGAAGAGTTACGR: GAGCAGGAAAGTGTTTTAGAA
*GAPDH* (human)	NM_002046	F: TGTGGTCATGAGTCCTTCCAR: CGAGATCCCTCCAAAATCAA
*CD80* (rat)	NM_012926	Rn00709368_m1
*CD86* (rat)	NM_020081	Rn00571654_m1
*CD163* (rat)	NM_001107887	Rn01492519_m1
*CD206* (rat)	NM_001106123	Rn01487342_m1
*NOS2* (rat)	NM_012611	Rn00561646_m1
*ARG1* (rat)	NM_017134	Rn00691090_m1
*HPRT1* (rat)	NM_012583	Rn01527840_m1

## Data Availability

The data presented in this study are all available in this article and the Supplementary Material.

## Supplementary Materials

The following supporting information can be downloaded at: https://www.mdpi.com/article/10.3390/ijms24087456/s1.
