# Supplementary material for: Mesenchymal Stem Cell Exosomes as Immunomodulatory Therapy for Corneal Scarring
Source: Int J Mol Sci. 2023 Apr 18;24(8):7456. doi: 10.3390/ijms24087456 (PMC10144287; doi:10.3390/ijms24087456)
Supplement: Supplementary file 1 [file ijms-24-07456-s001.zip › Supplementary Table S1.pdf]

Table S1. The number of rats used in experiments.

	Treatment group	
**Experiment**	**Epithelium-on**	**Epithelium-off**
Time-lapse exosome tracing	5	5
	**PBS**	**MSC-exo**
Corneal neovascularization assay	5	5
Whole-mount COL3A1, FN, and α-SMA immunofluorescence staining*	3	3
Frozen section CD80, CD86, CD163, and CD206 immunofluorescence staining*	3	3
RT-PCR of M1 and M2 macrophage markers*	3 x 3 = 9	3 x 3 = 9
ELISA and multiplex of cytokines/chemokines on day 2*	3 x 3 = 9	3 x 3 = 9
ELISA and multiplex of cytokines/chemokines on day 5*	3 x 3 = 9	3 x 3 = 9

*Naïve corneas of the fellow eyes were used as controls
