# Supplementary material for: Mesenchymal Stem Cell Exosomes as Immunomodulatory Therapy for Corneal Scarring
Source: Int J Mol Sci. 2023 Apr 18;24(8):7456. doi: 10.3390/ijms24087456 (PMC10144287; doi:10.3390/ijms24087456)
Supplement: Supplementary file 1 [file ijms-24-07456-s001.zip › Supplementary Table S2.pdf]

Table S2. Cytokine and chemokine concentrations (in ng/mg) in non-treated and MSC-exo-treated corneas.

	Day 2				Day 5			
	**Control**	**PBS**	**MSc-Exo**	**p value (PBS vs. MSc-Exo**	**Control**	**PBS**	**MSc-Exo**	**p value (PBS vs. MSc-Exo)**
IL-1β	7.3±2.1	301.8±145.8	138.3±63.4	0.016	4.0±0.5	116.5±22.5	87.8±30.3	0.183
IL-8	0.5±0.1	64.0±13.2	36.0±13.3	0.002	0.6±0.1	24.0±23.8	21.5±12.0	0.854
TNF-α	1.8±1.1	4.3±1.9	2.7±1.2	0.042	0.7±0.1	3.2±0.9	2.7±0.8	0.305
IL-10	0.7±0.4	2.1±1.8	3.2±1.5	0.039	0.6±0.1	1.7±0.9	1.8±0.7	0.941
CXCL1	62.7±18.4	1031.3±268.9	1001.1±216.0	0.809	60.3±9.3	832.5±164.0	671.3±110.3	0.020
α-MPO	127.0±37.3	273.4±48.3	278.7±19.3	0.186	94.1±9.6	253.1±22.6	214.3±27.4	0.001
